# Supplementary material for: Niche Tet maintains germline stem cells independently of dioxygenase activity
Source: EMBO J. 2024 Mar 18;43(8):9. doi: 10.1038/s44318-024-00074-9 (PMC11021519; doi:10.1038/s44318-024-00074-9)
Supplement: Supplementary file 1 — Appendix [file 44318_2024_74_MOESM1_ESM.pdf]

Table of Content

Appendix Figure S1 - Validating RNAi efficiency with qPCR. (Page 2)

Appendix Figure S2 - Stat92E is required in the niche to promote dpp expression and maintain GSCs. (Page 3)

Appendix Figure S1

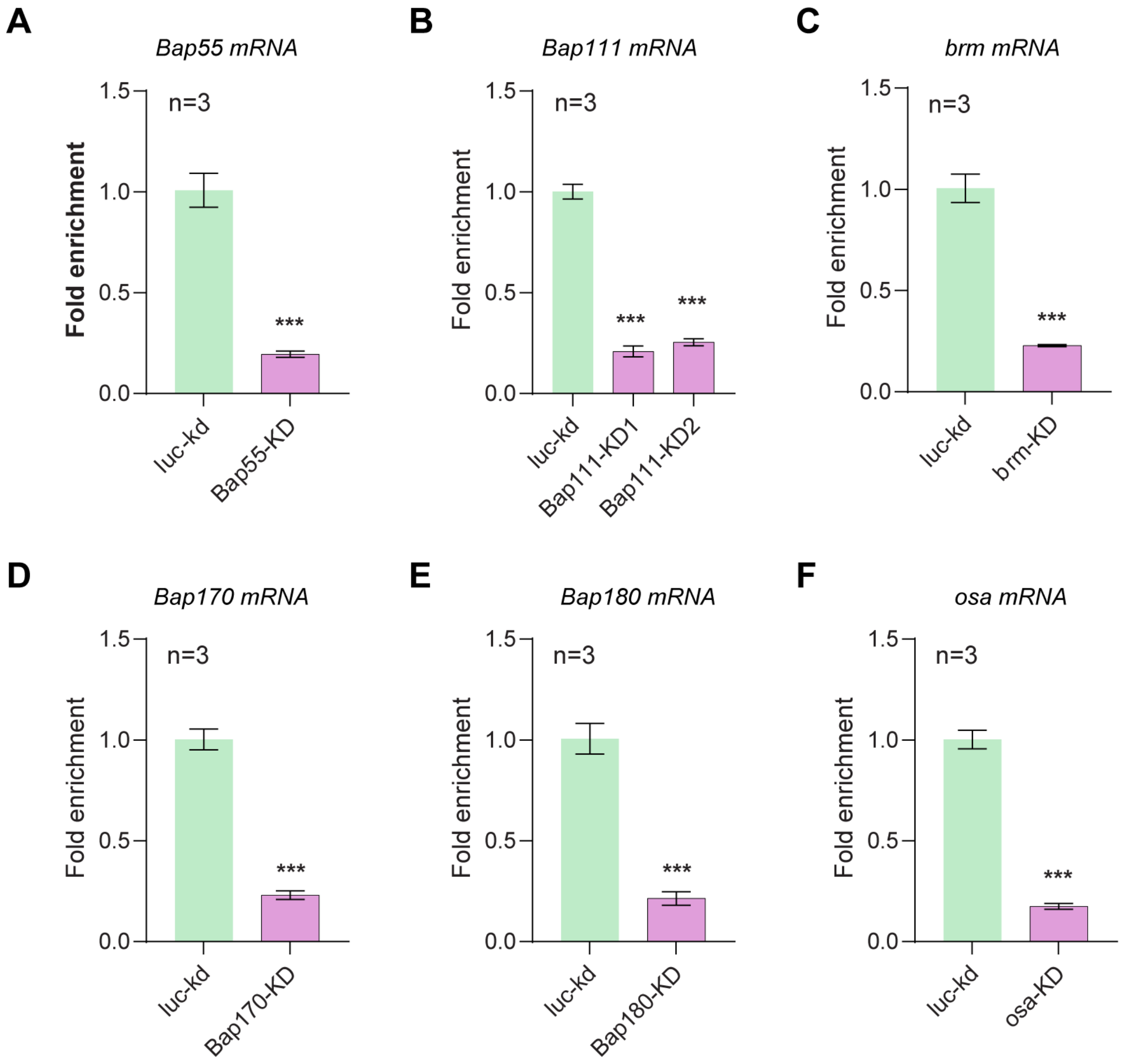

Appendix Figure S1 - Validating RNAi efficiency with qPCR**.**

tub-Gal4^{ts}-driven shRNA expression can efficiently knock down *Bap55* **(A)**, *Bap111* **(B)**, *brm* **(C)**, *Bap170* **(D)**, *Bap180* **(E)** and *osa* **(F)** mRNAs in adult flies.

Data information: In (A-F), data are presented as mean ± SEM. ***P≤0.001 (Student's t-test). In (B-F), n = number of technical replicates.

Appendix Figure S2

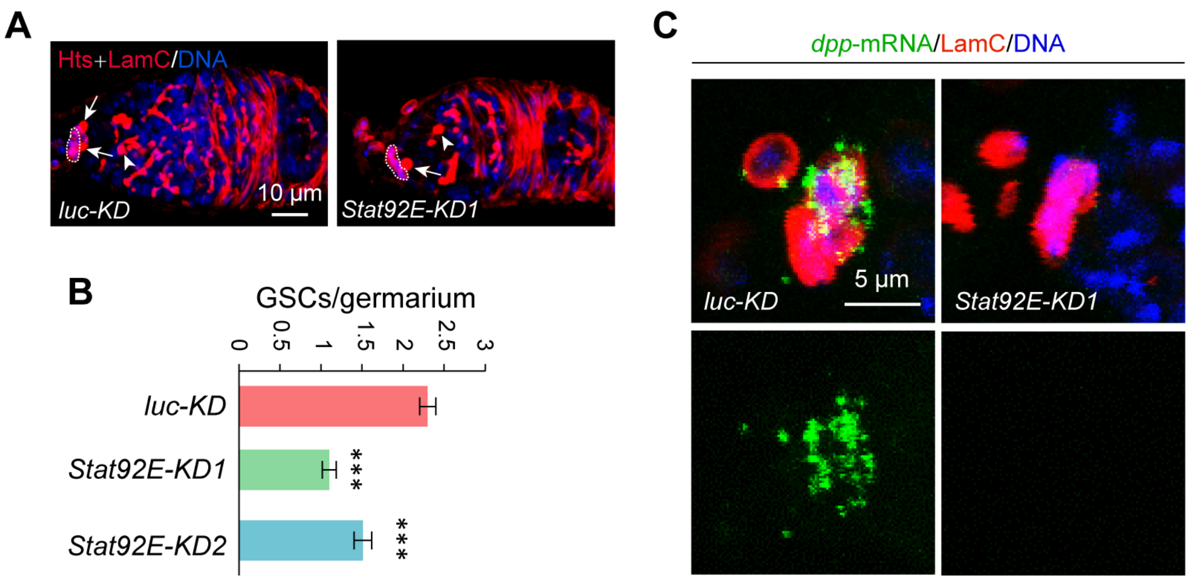

Appendix Figure S2 - Stat92E is required in the niche to promote *dpp* expression and maintain GSCs.

A-B *bab1^{ts}*-driven Stat92E knockdown has no obvious effect on cap cell but causes GSC loss. **B**: quantification results.

C *bab1^{ts}*-driven *Stat92E* knockdown dramatically decreases *dpp* mRNA expression in cap cells (n = number of germaria). Data are presented as mean ± SEM. ***P≤0.001 (Student's t-test).
