# Supplementary figures and images for: Niche Tet maintains germline stem cells independently of dioxygenase activity (part 1 of 2)
Source: EMBO J. 2024 Mar 18;43(8):9. doi: 10.1038/s44318-024-00074-9 (PMC11021519; doi:10.1038/s44318-024-00074-9)

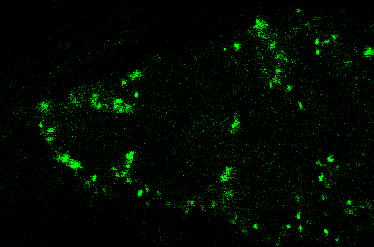

Supplement: Supplementary file 2 — Source Data Fig. 1 [file 44318_2024_74_MOESM2_ESM.zip › 1A/1A_Bottom_Left.tif]

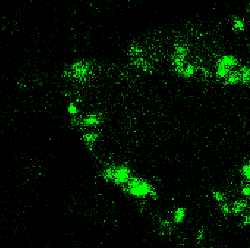

Supplement: Supplementary file 2 — Source Data Fig. 1 [file 44318_2024_74_MOESM2_ESM.zip › 1A/1A_Bottom_Right.tif]

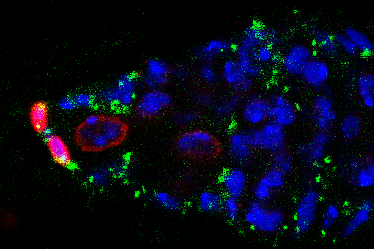

Supplement: Supplementary file 2 — Source Data Fig. 1 [file 44318_2024_74_MOESM2_ESM.zip › 1A/1A_Top_Left.tif]

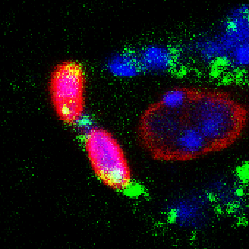

Supplement: Supplementary file 2 — Source Data Fig. 1 [file 44318_2024_74_MOESM2_ESM.zip › 1A/1A_Top_Right.tif]

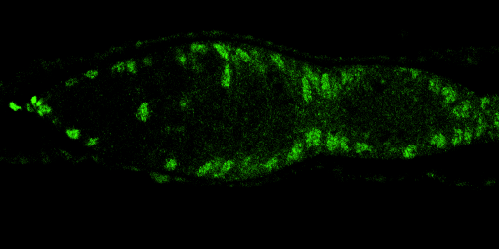

Supplement: Supplementary file 2 — Source Data Fig. 1 [file 44318_2024_74_MOESM2_ESM.zip › 1B/1B_Bottom_Left.tif]

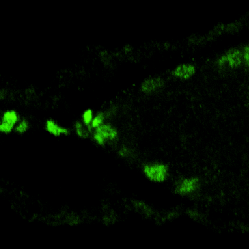

Supplement: Supplementary file 2 — Source Data Fig. 1 [file 44318_2024_74_MOESM2_ESM.zip › 1B/1B_Bottom_Right.tif]

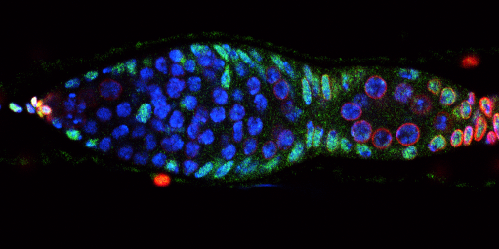

Supplement: Supplementary file 2 — Source Data Fig. 1 [file 44318_2024_74_MOESM2_ESM.zip › 1B/1B_Top_Left.tif]

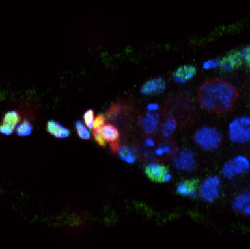

Supplement: Supplementary file 2 — Source Data Fig. 1 [file 44318_2024_74_MOESM2_ESM.zip › 1B/1B_Top_Right.tif]

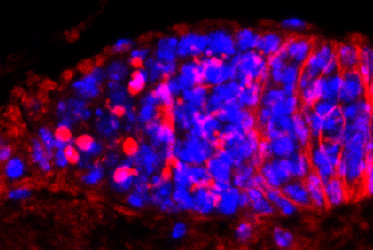

Supplement: Supplementary file 2 — Source Data Fig. 1 [file 44318_2024_74_MOESM2_ESM.zip › 1C/1C_luc-KD.tif]

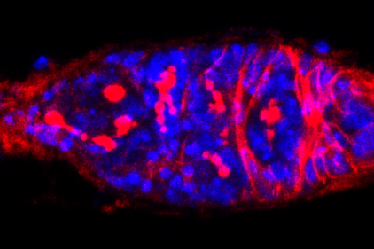

Supplement: Supplementary file 2 — Source Data Fig. 1 [file 44318_2024_74_MOESM2_ESM.zip › 1C/1C_Tet-KD1.tif]

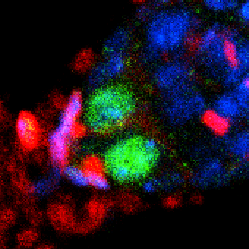

Supplement: Supplementary file 2 — Source Data Fig. 1 [file 44318_2024_74_MOESM2_ESM.zip › 1E/1E_luc-KD.tif]

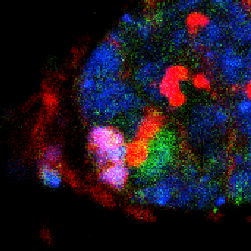

Supplement: Supplementary file 2 — Source Data Fig. 1 [file 44318_2024_74_MOESM2_ESM.zip › 1E/1E_Tet-KD1.tif]

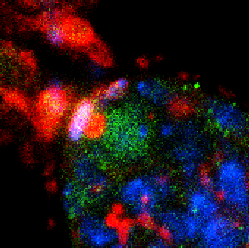

Supplement: Supplementary file 2 — Source Data Fig. 1 [file 44318_2024_74_MOESM2_ESM.zip › 1E/1E_Tet-KD2.tif]

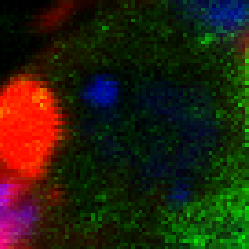

Supplement: Supplementary file 2 — Source Data Fig. 1 [file 44318_2024_74_MOESM2_ESM.zip › 1G/1G_luc-KD_Bottom.tif]

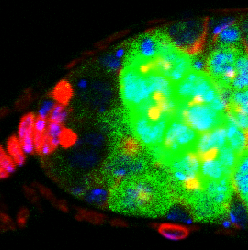

Supplement: Supplementary file 2 — Source Data Fig. 1 [file 44318_2024_74_MOESM2_ESM.zip › 1G/1G_luc-KD_Top.tif]

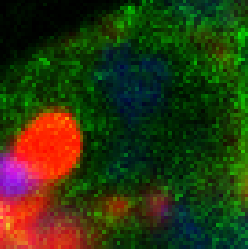

Supplement: Supplementary file 2 — Source Data Fig. 1 [file 44318_2024_74_MOESM2_ESM.zip › 1G/1G_Tet-KD1_Bottom.tif]

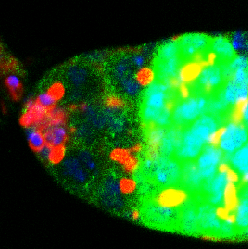

Supplement: Supplementary file 2 — Source Data Fig. 1 [file 44318_2024_74_MOESM2_ESM.zip › 1G/1G_Tet-KD1_Top.tif]

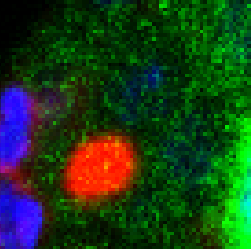

Supplement: Supplementary file 2 — Source Data Fig. 1 [file 44318_2024_74_MOESM2_ESM.zip › 1G/1G_Tet-KD2_Bottom.tif]

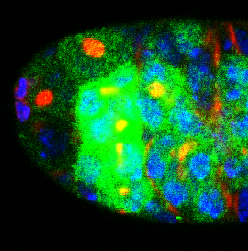

Supplement: Supplementary file 2 — Source Data Fig. 1 [file 44318_2024_74_MOESM2_ESM.zip › 1G/1G_Tet-KD2_Top.tif]

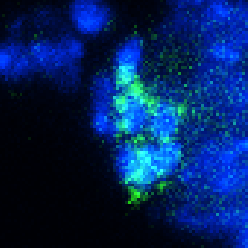

Supplement: Supplementary file 2 — Source Data Fig. 1 [file 44318_2024_74_MOESM2_ESM.zip › 1I/1I_luc-KD.tif]

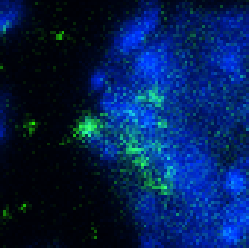

Supplement: Supplementary file 2 — Source Data Fig. 1 [file 44318_2024_74_MOESM2_ESM.zip › 1I/1I_Tet-KD1.tif]

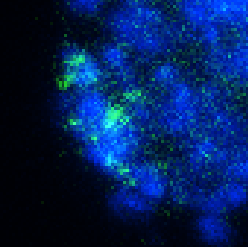

Supplement: Supplementary file 2 — Source Data Fig. 1 [file 44318_2024_74_MOESM2_ESM.zip › 1I/1I_Tet-KD2.tif]

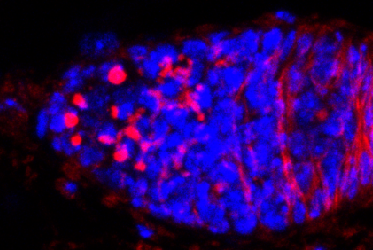

Supplement: Supplementary file 2 — Source Data Fig. 1 [file 44318_2024_74_MOESM2_ESM.zip › 1K/1K_dpp rescue.tif]

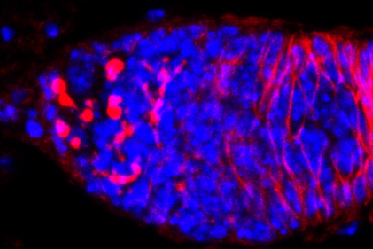

Supplement: Supplementary file 2 — Source Data Fig. 1 [file 44318_2024_74_MOESM2_ESM.zip › 1K/1K_luc-KD.tif]

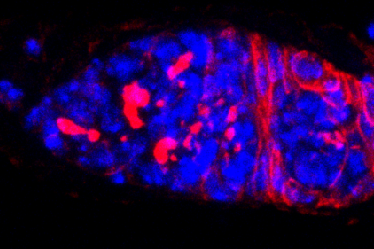

Supplement: Supplementary file 2 — Source Data Fig. 1 [file 44318_2024_74_MOESM2_ESM.zip › 1K/1K_Tet-KD1.tif]

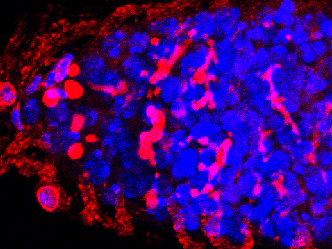

Supplement: Supplementary file 3 — Source Data Fig. 2 [file 44318_2024_74_MOESM3_ESM.zip › 2B/2B_luc-KD.tif]

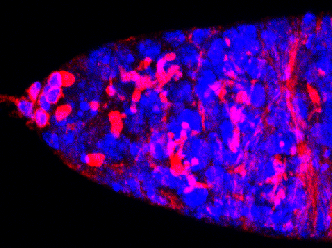

Supplement: Supplementary file 3 — Source Data Fig. 2 [file 44318_2024_74_MOESM3_ESM.zip › 2B/2B_TetED-OE.tif]

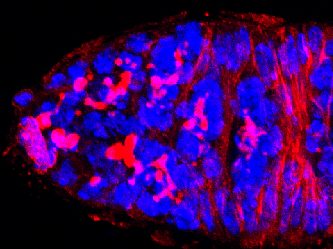

Supplement: Supplementary file 3 — Source Data Fig. 2 [file 44318_2024_74_MOESM3_ESM.zip › 2B/2B_TetED-Res.tif]

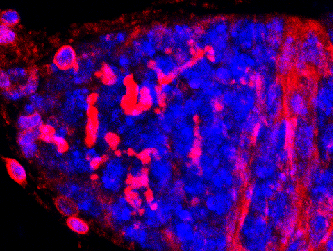

Supplement: Supplementary file 3 — Source Data Fig. 2 [file 44318_2024_74_MOESM3_ESM.zip › 2B/2B_Tet-KD1.tif]

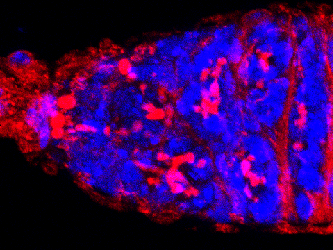

Supplement: Supplementary file 3 — Source Data Fig. 2 [file 44318_2024_74_MOESM3_ESM.zip › 2B/2B_TetWT-OE.tif]

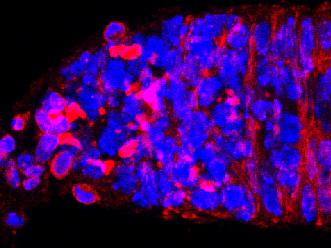

Supplement: Supplementary file 3 — Source Data Fig. 2 [file 44318_2024_74_MOESM3_ESM.zip › 2B/2B_TetWT-Res.tif]

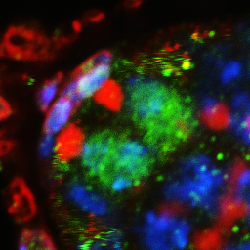

Supplement: Supplementary file 3 — Source Data Fig. 2 [file 44318_2024_74_MOESM3_ESM.zip › 2D/2D_luc-KD.tif]

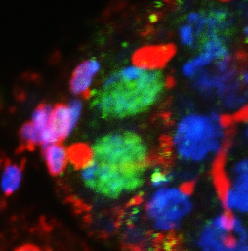

Supplement: Supplementary file 3 — Source Data Fig. 2 [file 44318_2024_74_MOESM3_ESM.zip › 2D/2D_TetED-OE.tif]

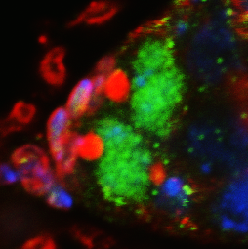

Supplement: Supplementary file 3 — Source Data Fig. 2 [file 44318_2024_74_MOESM3_ESM.zip › 2D/2D_TetED-Res.tif]

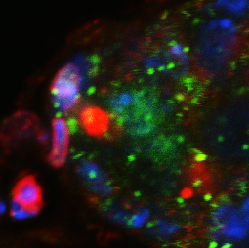

Supplement: Supplementary file 3 — Source Data Fig. 2 [file 44318_2024_74_MOESM3_ESM.zip › 2D/2D_Tet-KD1.tif]

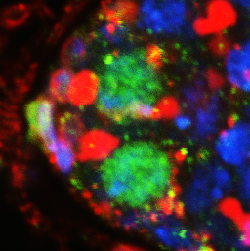

Supplement: Supplementary file 3 — Source Data Fig. 2 [file 44318_2024_74_MOESM3_ESM.zip › 2D/2D_TetWT-OE.tif]

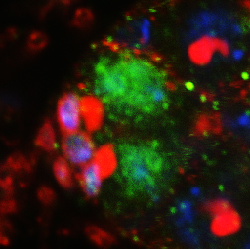

Supplement: Supplementary file 3 — Source Data Fig. 2 [file 44318_2024_74_MOESM3_ESM.zip › 2D/2D_TetWT-Res.tif]

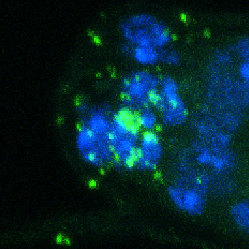

Supplement: Supplementary file 3 — Source Data Fig. 2 [file 44318_2024_74_MOESM3_ESM.zip › 2F/2F_luc-KD.tif]

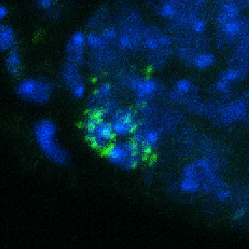

Supplement: Supplementary file 3 — Source Data Fig. 2 [file 44318_2024_74_MOESM3_ESM.zip › 2F/2F_TetED-OE.tif]

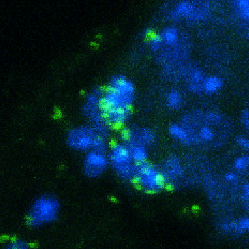

Supplement: Supplementary file 3 — Source Data Fig. 2 [file 44318_2024_74_MOESM3_ESM.zip › 2F/2F_TetED-Res.tif]

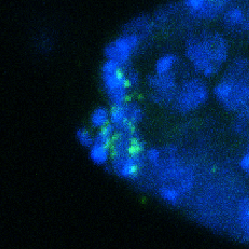

Supplement: Supplementary file 3 — Source Data Fig. 2 [file 44318_2024_74_MOESM3_ESM.zip › 2F/2F_Tet-KD1.tif]

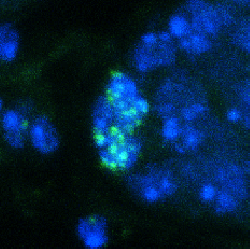

Supplement: Supplementary file 3 — Source Data Fig. 2 [file 44318_2024_74_MOESM3_ESM.zip › 2F/2F_TetWT-OE.tif]

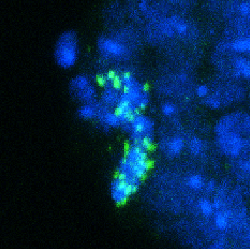

Supplement: Supplementary file 3 — Source Data Fig. 2 [file 44318_2024_74_MOESM3_ESM.zip › 2F/2F_TetWT-Res.tif]

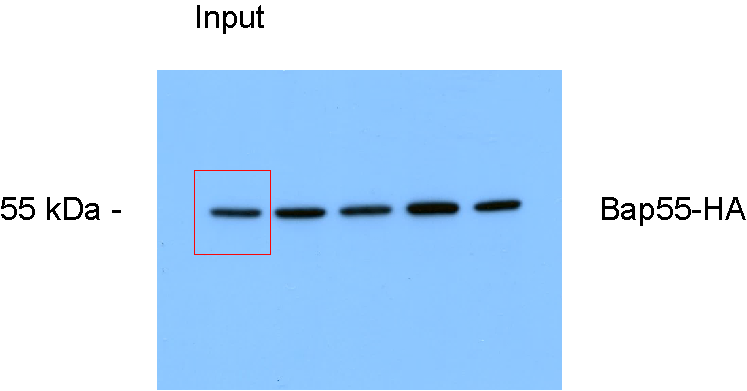

Supplement: Supplementary file 4 — Source Data Fig. 3 [file 44318_2024_74_MOESM4_ESM.zip › 3C/3C_Bottom_Left_Western_Bap55-HA.tif]

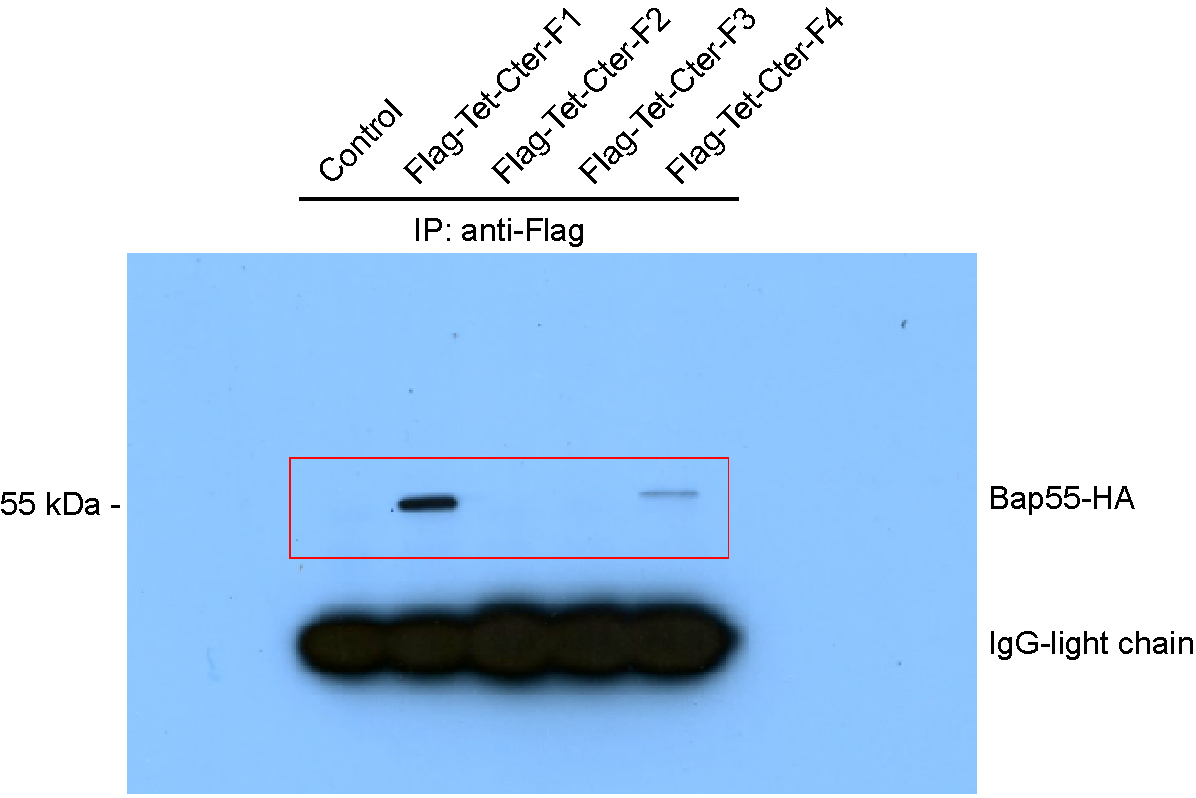

Supplement: Supplementary file 4 — Source Data Fig. 3 [file 44318_2024_74_MOESM4_ESM.zip › 3C/3C_Bottom_Right_Western_Bap55-HA.tif]

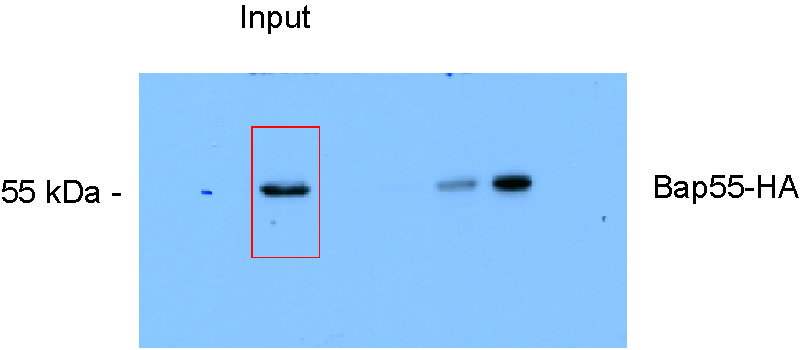

Supplement: Supplementary file 4 — Source Data Fig. 3 [file 44318_2024_74_MOESM4_ESM.zip › 3C/3C_Top_Left_Western_Bap55-HA.tif]

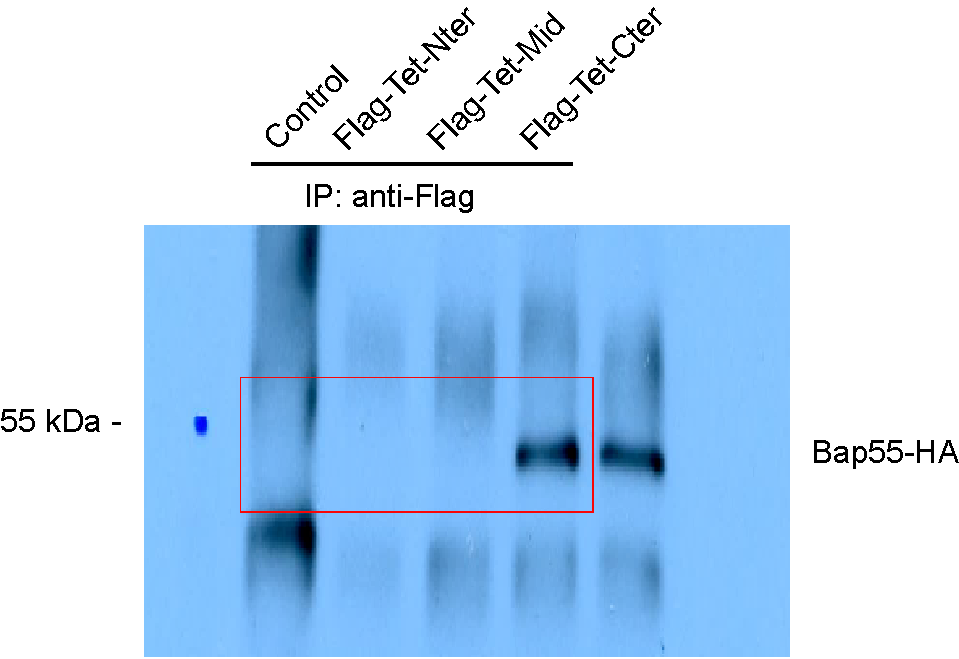

Supplement: Supplementary file 4 — Source Data Fig. 3 [file 44318_2024_74_MOESM4_ESM.zip › 3C/3C_Top_Right_Western_Bap55-HA.tif]

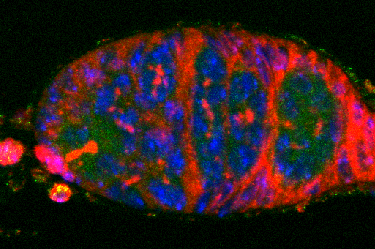

Supplement: Supplementary file 4 — Source Data Fig. 3 [file 44318_2024_74_MOESM4_ESM.zip › 3D/3D_Bap111-KD1.tif]

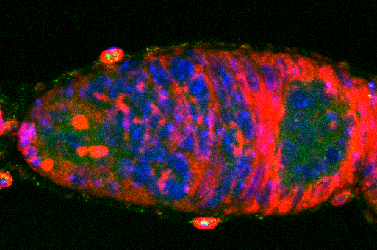

Supplement: Supplementary file 4 — Source Data Fig. 3 [file 44318_2024_74_MOESM4_ESM.zip › 3D/3D_Bap55-KD.tif]

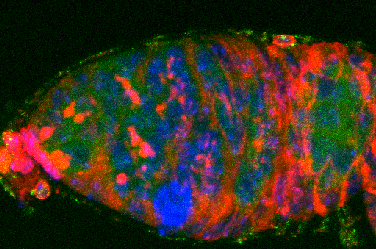

Supplement: Supplementary file 4 — Source Data Fig. 3 [file 44318_2024_74_MOESM4_ESM.zip › 3D/3D_luc-KD.tif]

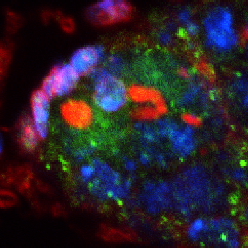

Supplement: Supplementary file 4 — Source Data Fig. 3 [file 44318_2024_74_MOESM4_ESM.zip › 3F/3F_Bap111-KD1.tif]

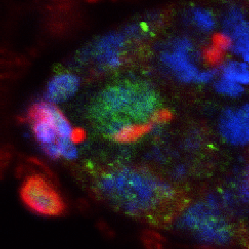

Supplement: Supplementary file 4 — Source Data Fig. 3 [file 44318_2024_74_MOESM4_ESM.zip › 3F/3F_Bap55-KD.tif]

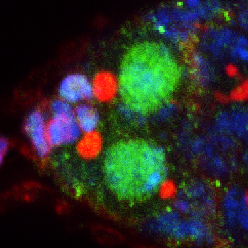

Supplement: Supplementary file 4 — Source Data Fig. 3 [file 44318_2024_74_MOESM4_ESM.zip › 3F/3F_luc-KD.tif]

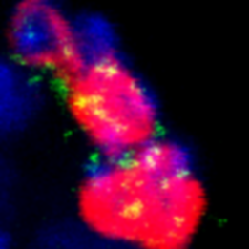

Supplement: Supplementary file 4 — Source Data Fig. 3 [file 44318_2024_74_MOESM4_ESM.zip › 3H/3H_Bap111-KD1.tif]

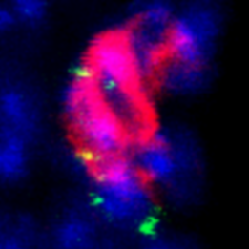

Supplement: Supplementary file 4 — Source Data Fig. 3 [file 44318_2024_74_MOESM4_ESM.zip › 3H/3H_Bap55-KD.tif]

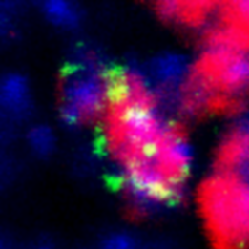

Supplement: Supplementary file 4 — Source Data Fig. 3 [file 44318_2024_74_MOESM4_ESM.zip › 3H/3H_luc-KD.tif]

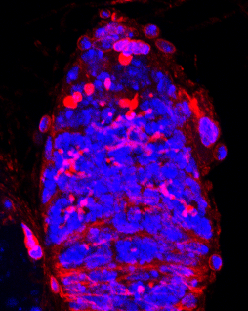

Supplement: Supplementary file 5 — Source Data Fig. 4 [file 44318_2024_74_MOESM5_ESM.zip › 4A/4A_Bap170-KD.tif]

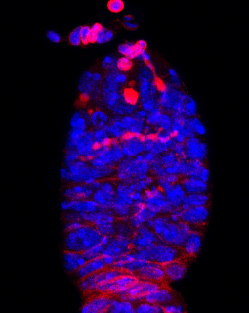

Supplement: Supplementary file 5 — Source Data Fig. 4 [file 44318_2024_74_MOESM5_ESM.zip › 4A/4A_Bap180-KD.tif]

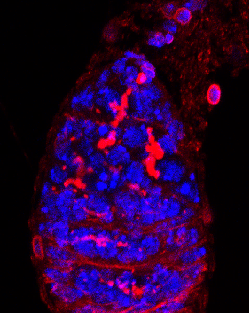

Supplement: Supplementary file 5 — Source Data Fig. 4 [file 44318_2024_74_MOESM5_ESM.zip › 4A/4A_brm-KD.tif]

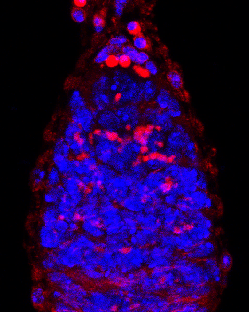

Supplement: Supplementary file 5 — Source Data Fig. 4 [file 44318_2024_74_MOESM5_ESM.zip › 4A/4A_luc-KD.tif]

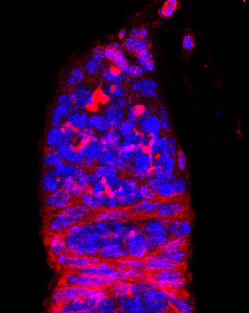

Supplement: Supplementary file 5 — Source Data Fig. 4 [file 44318_2024_74_MOESM5_ESM.zip › 4A/4A_osa-KD.tif]

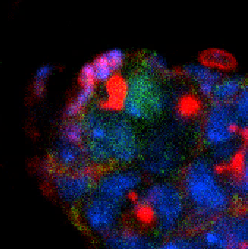

Supplement: Supplementary file 5 — Source Data Fig. 4 [file 44318_2024_74_MOESM5_ESM.zip › 4C/4C_Bap170-KD.tif]

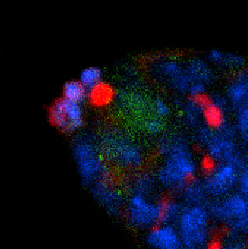

Supplement: Supplementary file 5 — Source Data Fig. 4 [file 44318_2024_74_MOESM5_ESM.zip › 4C/4C_Bap180-KD.tif]

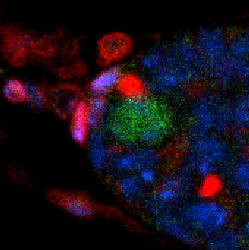

Supplement: Supplementary file 5 — Source Data Fig. 4 [file 44318_2024_74_MOESM5_ESM.zip › 4C/4C_brm-KD.tif]

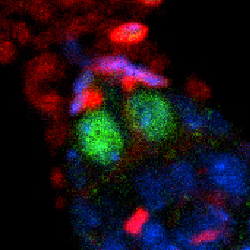

Supplement: Supplementary file 5 — Source Data Fig. 4 [file 44318_2024_74_MOESM5_ESM.zip › 4C/4C_luc-KD.tif]

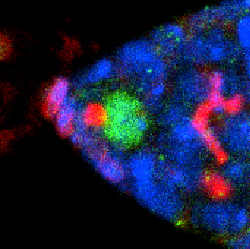

Supplement: Supplementary file 5 — Source Data Fig. 4 [file 44318_2024_74_MOESM5_ESM.zip › 4C/4C_osa-KD.tif]

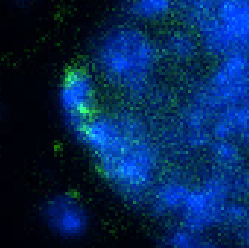

Supplement: Supplementary file 5 — Source Data Fig. 4 [file 44318_2024_74_MOESM5_ESM.zip › 4E/4E_Bap170-KD.tif]

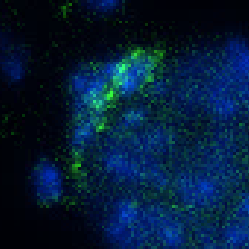

Supplement: Supplementary file 5 — Source Data Fig. 4 [file 44318_2024_74_MOESM5_ESM.zip › 4E/4E_Bap180-KD.tif]

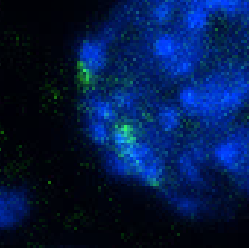

Supplement: Supplementary file 5 — Source Data Fig. 4 [file 44318_2024_74_MOESM5_ESM.zip › 4E/4E_brm-KD.tif]

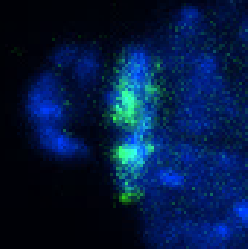

Supplement: Supplementary file 5 — Source Data Fig. 4 [file 44318_2024_74_MOESM5_ESM.zip › 4E/4E_luc-KD.tif]

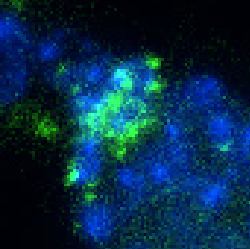

Supplement: Supplementary file 5 — Source Data Fig. 4 [file 44318_2024_74_MOESM5_ESM.zip › 4E/4E_osa-KD.tif]

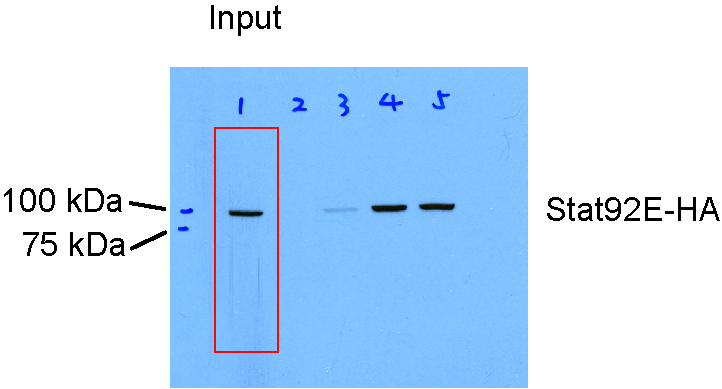

Supplement: Supplementary file 6 — Source Data Fig. 5 [file 44318_2024_74_MOESM6_ESM.zip › 5A/5A_Left.tif]

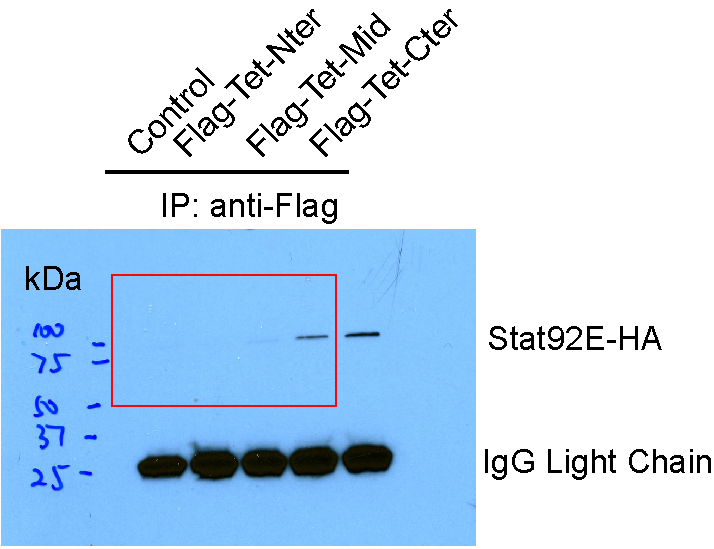

Supplement: Supplementary file 6 — Source Data Fig. 5 [file 44318_2024_74_MOESM6_ESM.zip › 5A/5A_Right.tif]

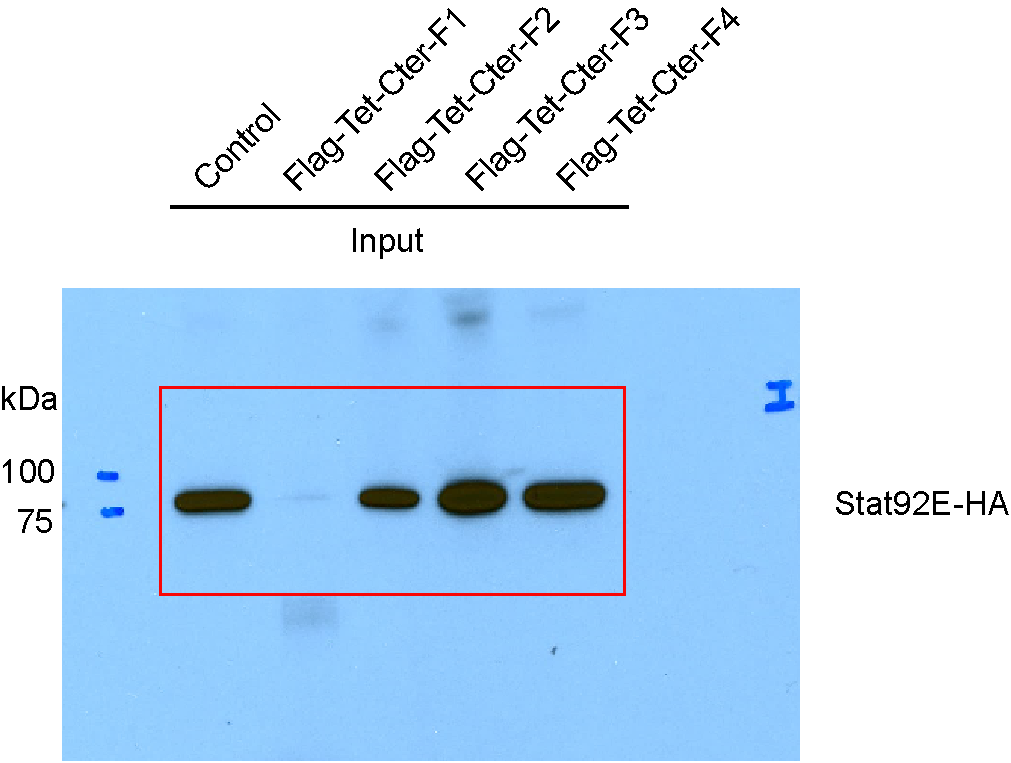

Supplement: Supplementary file 6 — Source Data Fig. 5 [file 44318_2024_74_MOESM6_ESM.zip › 5B/5B_Bottom_Left.tif]

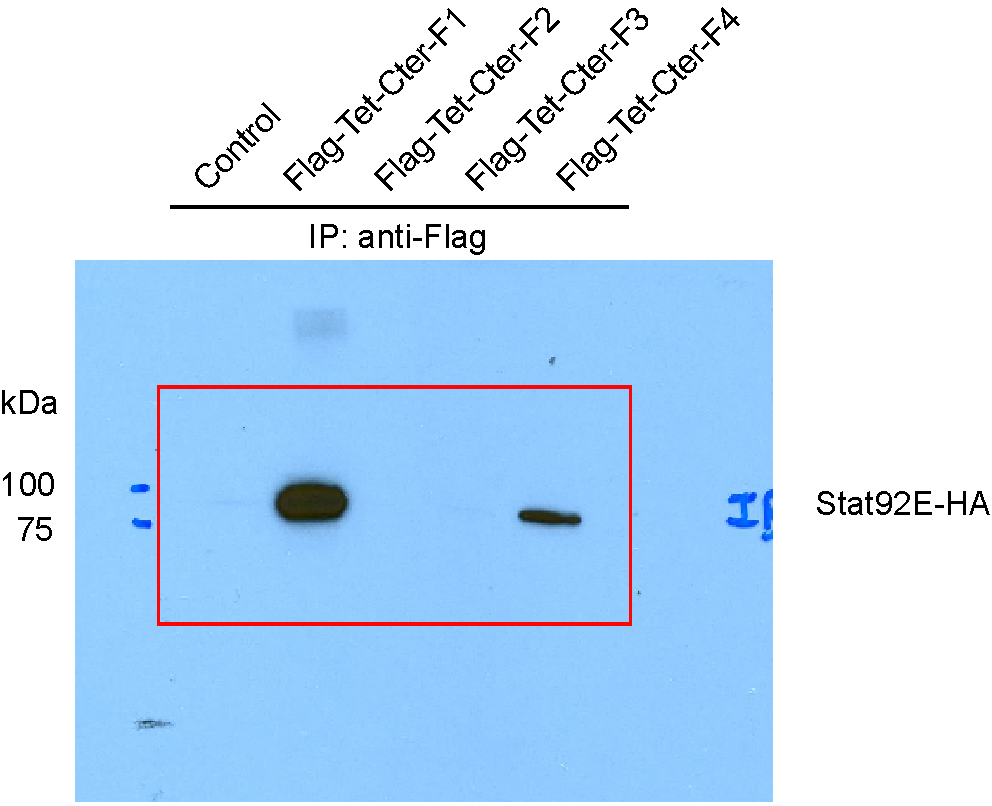

Supplement: Supplementary file 6 — Source Data Fig. 5 [file 44318_2024_74_MOESM6_ESM.zip › 5B/5B_Bottom_Right.tif]

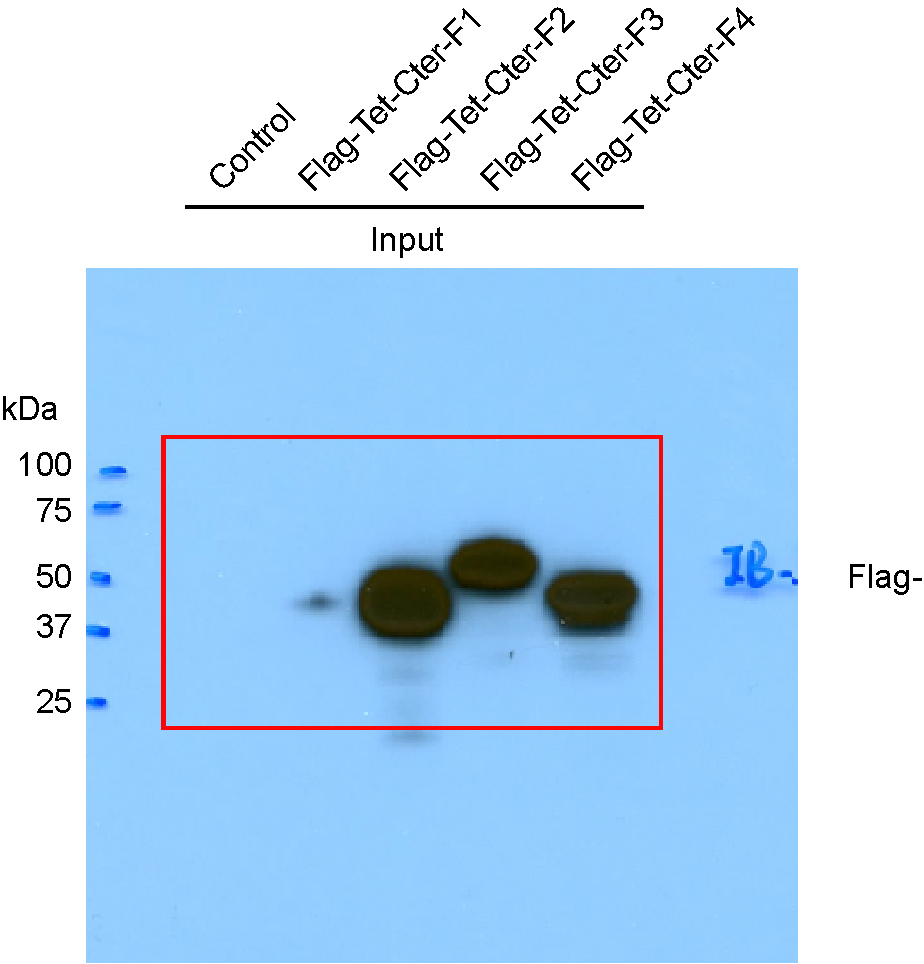

Supplement: Supplementary file 6 — Source Data Fig. 5 [file 44318_2024_74_MOESM6_ESM.zip › 5B/5B_Top_Left.tif]

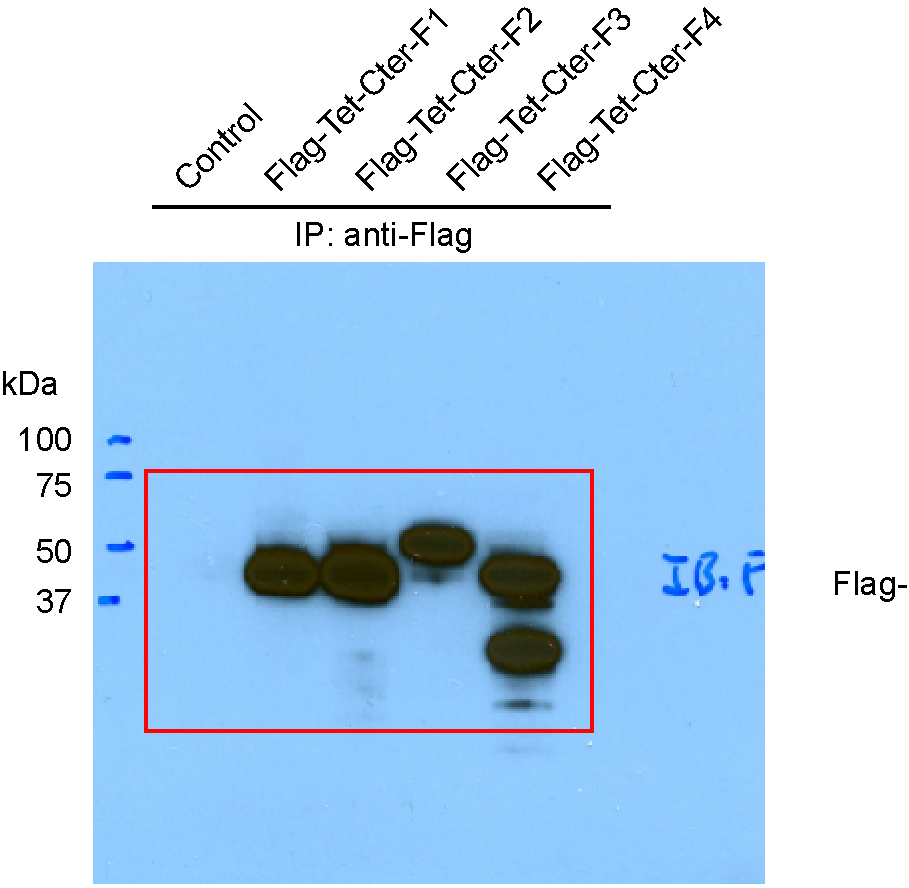

Supplement: Supplementary file 6 — Source Data Fig. 5 [file 44318_2024_74_MOESM6_ESM.zip › 5B/5B_Top_Right.tif]

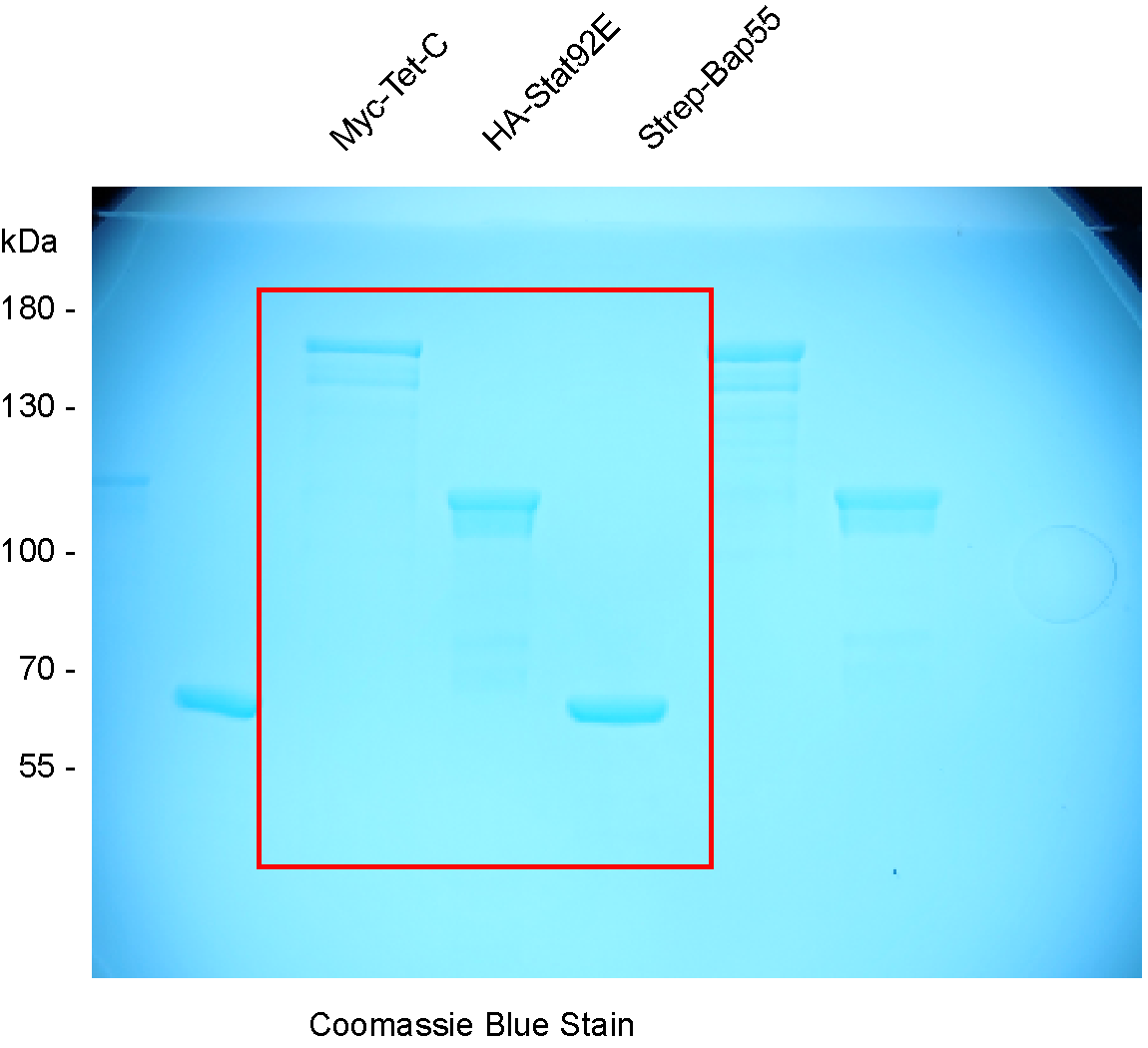

Supplement: Supplementary file 6 — Source Data Fig. 5 [file 44318_2024_74_MOESM6_ESM.zip › 5C/5C.tif]

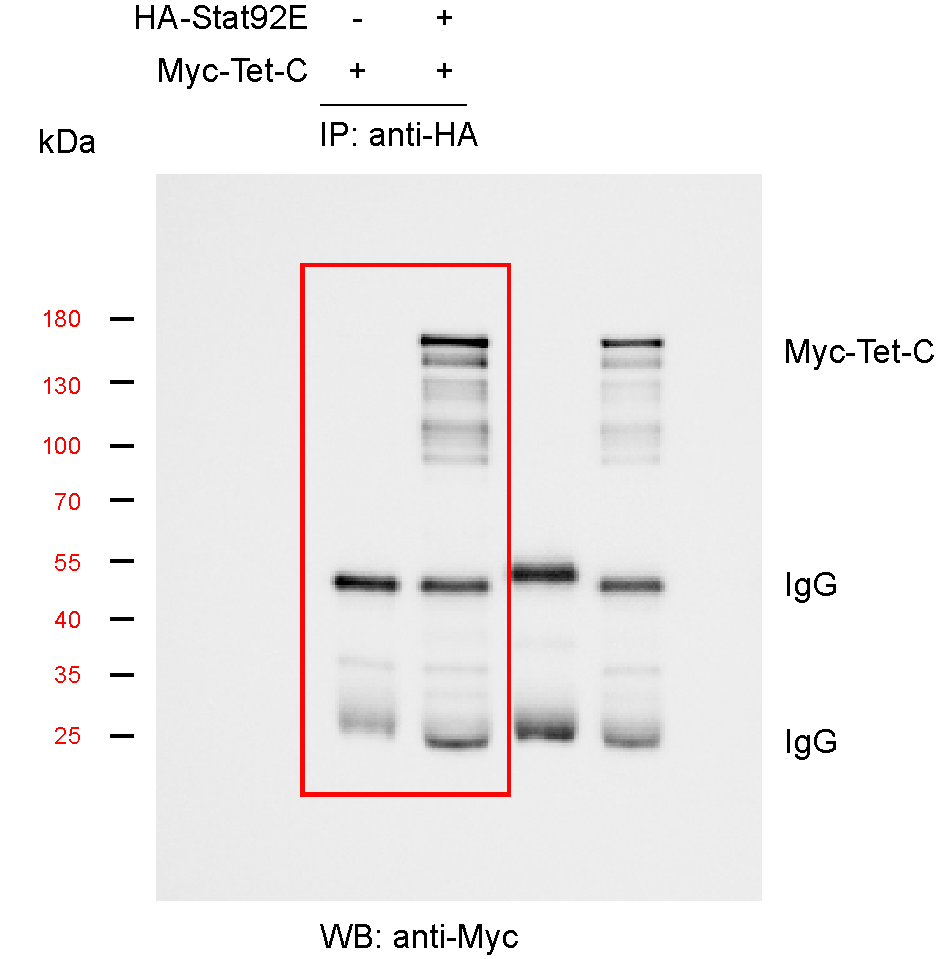

Supplement: Supplementary file 6 — Source Data Fig. 5 [file 44318_2024_74_MOESM6_ESM.zip › 5D/5D.tif]

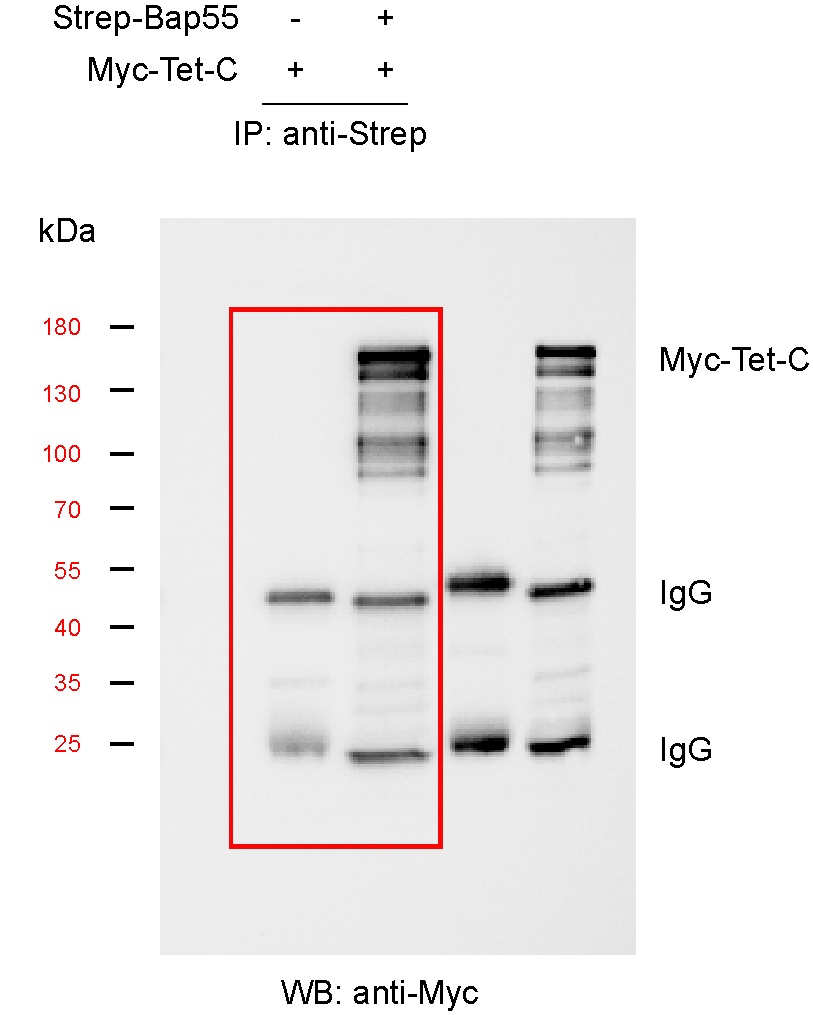

Supplement: Supplementary file 6 — Source Data Fig. 5 [file 44318_2024_74_MOESM6_ESM.zip › 5E/5E.tif]

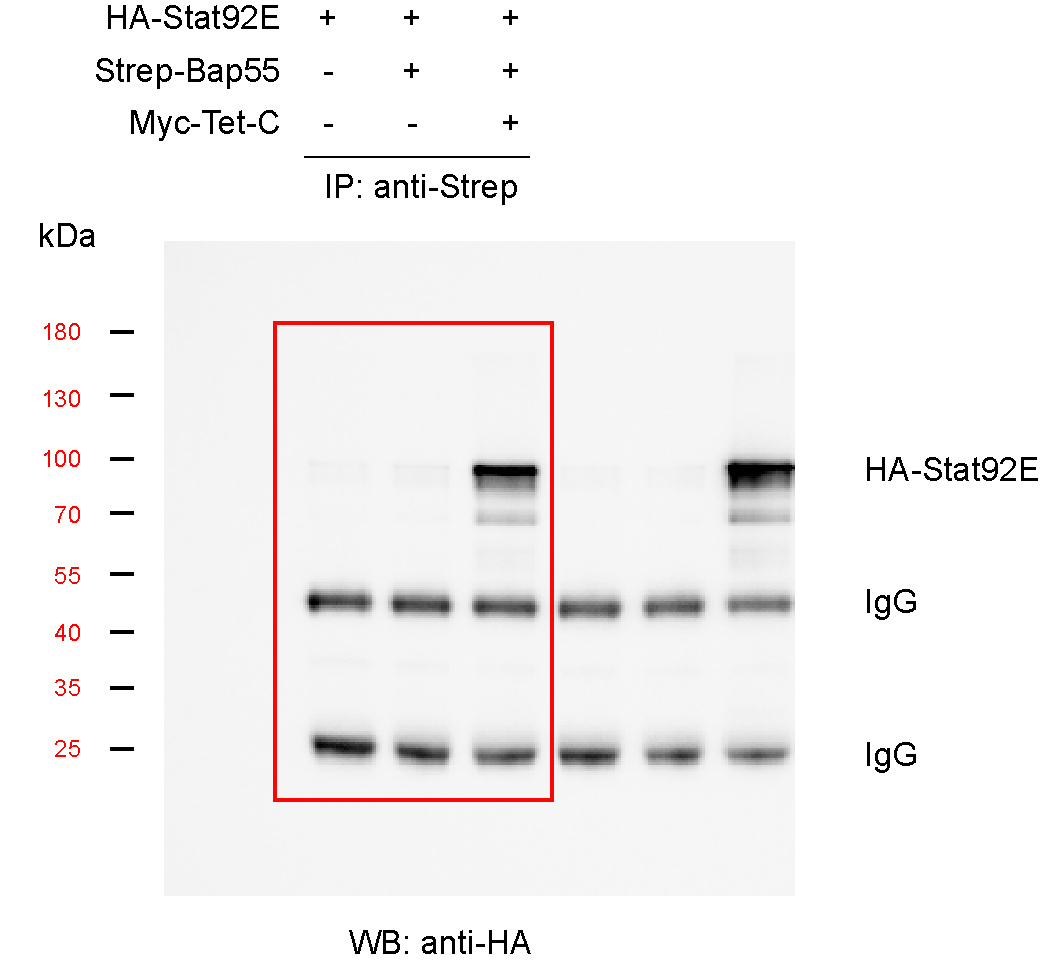

Supplement: Supplementary file 6 — Source Data Fig. 5 [file 44318_2024_74_MOESM6_ESM.zip › 5F/5F.tif]

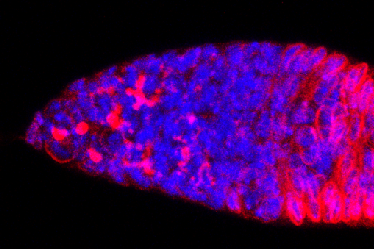

Supplement: Supplementary file 6 — Source Data Fig. 5 [file 44318_2024_74_MOESM6_ESM.zip › 5G/5G_Bap55_Res.tif]

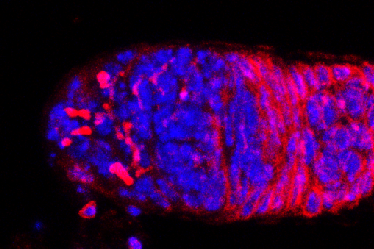

Supplement: Supplementary file 6 — Source Data Fig. 5 [file 44318_2024_74_MOESM6_ESM.zip › 5G/5G_Bap55-Stat92E-Res.tif]

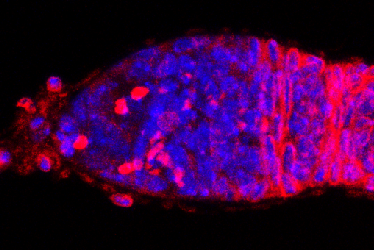

Supplement: Supplementary file 6 — Source Data Fig. 5 [file 44318_2024_74_MOESM6_ESM.zip › 5G/5G_Stat92E_Res.tif]

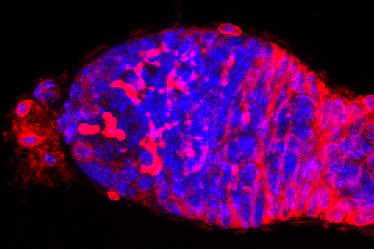

Supplement: Supplementary file 6 — Source Data Fig. 5 [file 44318_2024_74_MOESM6_ESM.zip › 5G/5G_Tet-KD1.tif]

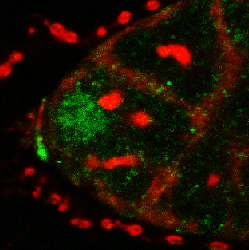

Supplement: Supplementary file 6 — Source Data Fig. 5 [file 44318_2024_74_MOESM6_ESM.zip › 5I/5I_Bap55_Res.tif]

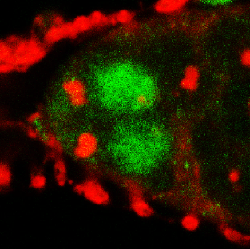

Supplement: Supplementary file 6 — Source Data Fig. 5 [file 44318_2024_74_MOESM6_ESM.zip › 5I/5I_Bap55-Stat92E-Res.tif]

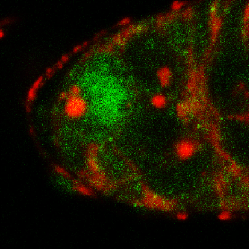

Supplement: Supplementary file 6 — Source Data Fig. 5 [file 44318_2024_74_MOESM6_ESM.zip › 5I/5I_Stat92E_Res.tif]

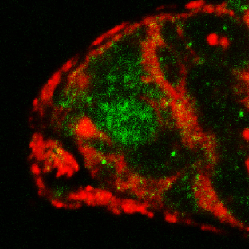

Supplement: Supplementary file 6 — Source Data Fig. 5 [file 44318_2024_74_MOESM6_ESM.zip › 5I/5I_Tet-KD1.tif]

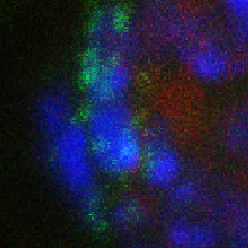

Supplement: Supplementary file 6 — Source Data Fig. 5 [file 44318_2024_74_MOESM6_ESM.zip › 5K/5K_Bap55_Res.tif]

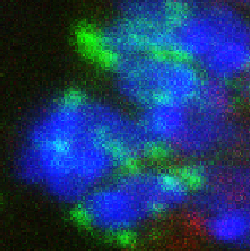

Supplement: Supplementary file 6 — Source Data Fig. 5 [file 44318_2024_74_MOESM6_ESM.zip › 5K/5K_Bap55-Stat92E-Res.tif]

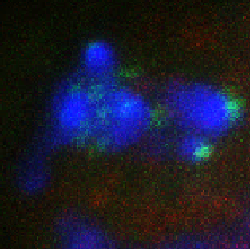

Supplement: Supplementary file 6 — Source Data Fig. 5 [file 44318_2024_74_MOESM6_ESM.zip › 5K/5K_Stat92E_Res.tif]

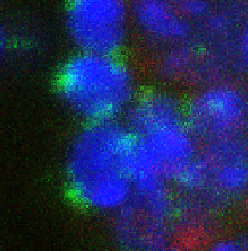

Supplement: Supplementary file 6 — Source Data Fig. 5 [file 44318_2024_74_MOESM6_ESM.zip › 5K/5K_Tet-KD1.tif]

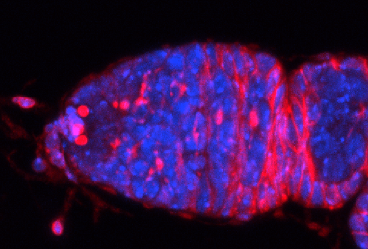

Supplement: Supplementary file 8 — Source Data Fig. 7 [file 44318_2024_74_MOESM8_ESM.zip › 7A/7A-hTET3-OE.tif]

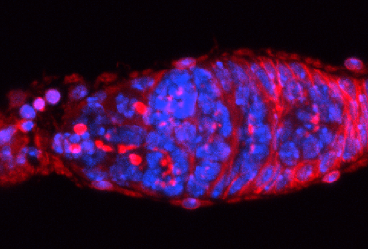

Supplement: Supplementary file 8 — Source Data Fig. 7 [file 44318_2024_74_MOESM8_ESM.zip › 7A/7A-hTET3-Res.tif]

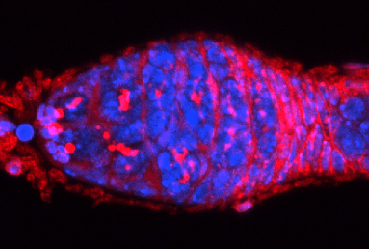

Supplement: Supplementary file 8 — Source Data Fig. 7 [file 44318_2024_74_MOESM8_ESM.zip › 7A/7A-luc-KD.tif]

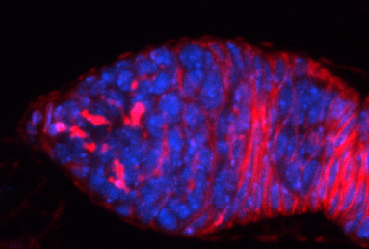

Supplement: Supplementary file 8 — Source Data Fig. 7 [file 44318_2024_74_MOESM8_ESM.zip › 7A/7A-Tet-KD1.tif]

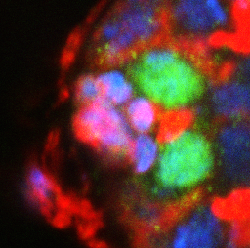

Supplement: Supplementary file 8 — Source Data Fig. 7 [file 44318_2024_74_MOESM8_ESM.zip › 7C/7C-hTET3-OE.tif]

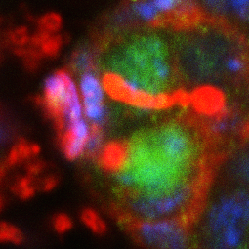

Supplement: Supplementary file 8 — Source Data Fig. 7 [file 44318_2024_74_MOESM8_ESM.zip › 7C/7C-hTET3-Res.tif]

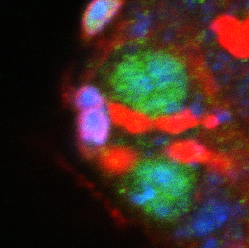

Supplement: Supplementary file 8 — Source Data Fig. 7 [file 44318_2024_74_MOESM8_ESM.zip › 7C/7C-luc-KD.tif]
