# Supplementary figures and images for: Niche Tet maintains germline stem cells independently of dioxygenase activity (part 2 of 2)
Source: EMBO J. 2024 Mar 18;43(8):9. doi: 10.1038/s44318-024-00074-9 (PMC11021519; doi:10.1038/s44318-024-00074-9)

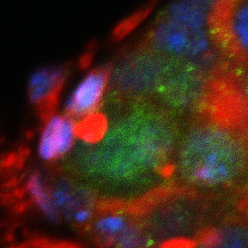

Supplement: Supplementary file 8 — Source Data Fig. 7 [file 44318_2024_74_MOESM8_ESM.zip › 7C/7C-Tet-KD1.tif]

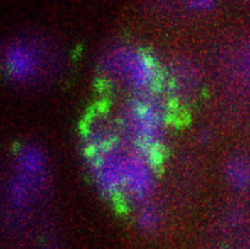

Supplement: Supplementary file 8 — Source Data Fig. 7 [file 44318_2024_74_MOESM8_ESM.zip › 7E/7E-hTET3-OE.tif]

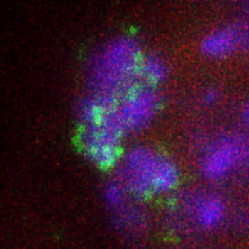

Supplement: Supplementary file 8 — Source Data Fig. 7 [file 44318_2024_74_MOESM8_ESM.zip › 7E/7E-hTET3-Res.tif]

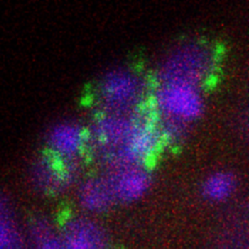

Supplement: Supplementary file 8 — Source Data Fig. 7 [file 44318_2024_74_MOESM8_ESM.zip › 7E/7E-luc-KD.tif]

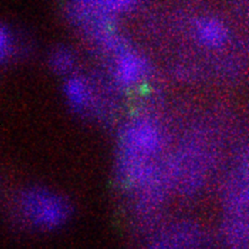

Supplement: Supplementary file 8 — Source Data Fig. 7 [file 44318_2024_74_MOESM8_ESM.zip › 7E/7E-Tet-KD1.tif]

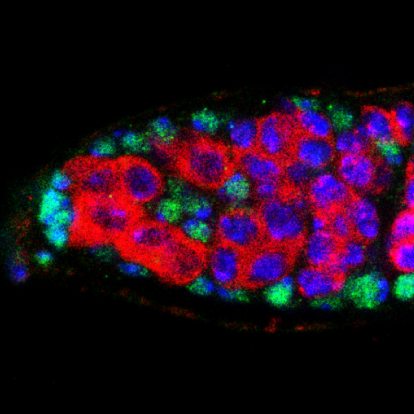

Supplement: Supplementary file 9 — EV Figure Source Data [file 44318_2024_74_MOESM9_ESM.zip › Figure EV1/EV1C/EV1C_Left.tif]

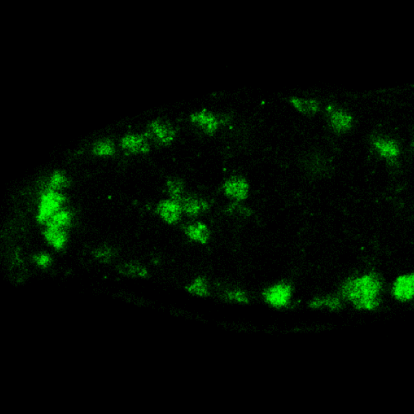

Supplement: Supplementary file 9 — EV Figure Source Data [file 44318_2024_74_MOESM9_ESM.zip › Figure EV1/EV1C/EV1C_Middle.tif]

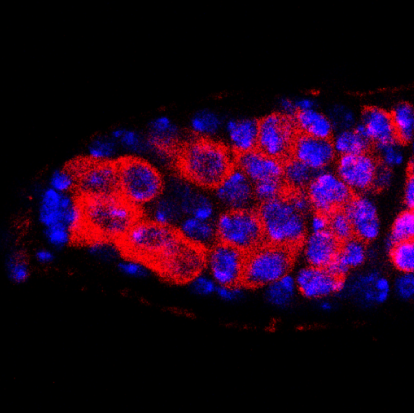

Supplement: Supplementary file 9 — EV Figure Source Data [file 44318_2024_74_MOESM9_ESM.zip › Figure EV1/EV1C/EV1C_Right.tif]

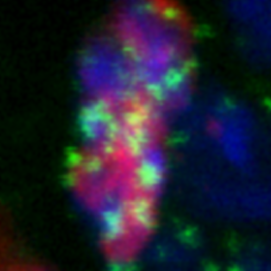

Supplement: Supplementary file 9 — EV Figure Source Data [file 44318_2024_74_MOESM9_ESM.zip › Figure EV1/EV1D/EV1D_luc-KD.tif]

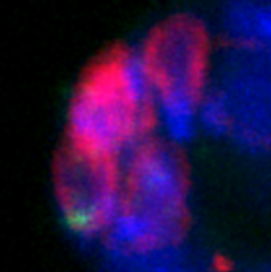

Supplement: Supplementary file 9 — EV Figure Source Data [file 44318_2024_74_MOESM9_ESM.zip › Figure EV1/EV1D/EV1D_Tet-KD1.tif]

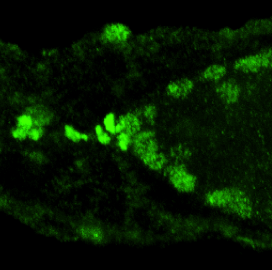

Supplement: Supplementary file 9 — EV Figure Source Data [file 44318_2024_74_MOESM9_ESM.zip › Figure EV1/EV1F/EV1F_luc-KD_Bottom.tif]

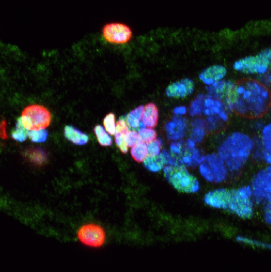

Supplement: Supplementary file 9 — EV Figure Source Data [file 44318_2024_74_MOESM9_ESM.zip › Figure EV1/EV1F/EV1F_luc-KD_Top.tif]

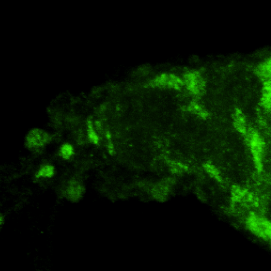

Supplement: Supplementary file 9 — EV Figure Source Data [file 44318_2024_74_MOESM9_ESM.zip › Figure EV1/EV1F/EV1F_Tet-KD1_Bottom.tif]

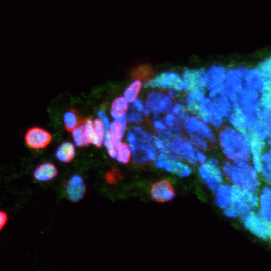

Supplement: Supplementary file 9 — EV Figure Source Data [file 44318_2024_74_MOESM9_ESM.zip › Figure EV1/EV1F/EV1F_Tet-KD1_Top.tif]

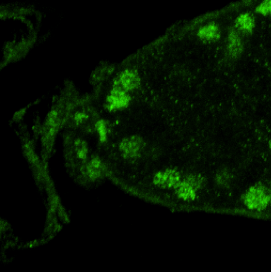

Supplement: Supplementary file 9 — EV Figure Source Data [file 44318_2024_74_MOESM9_ESM.zip › Figure EV1/EV1F/EV1F_Tet-KD2_Bottom.tif]

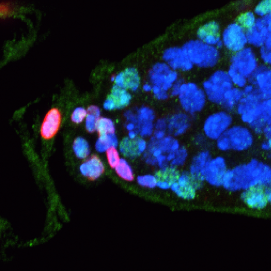

Supplement: Supplementary file 9 — EV Figure Source Data [file 44318_2024_74_MOESM9_ESM.zip › Figure EV1/EV1F/EV1F_Tet-KD2_Top.tif]

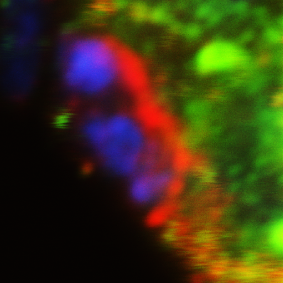

Supplement: Supplementary file 9 — EV Figure Source Data [file 44318_2024_74_MOESM9_ESM.zip › Figure EV2/EV2A/EV2A_luc-KD.tif]

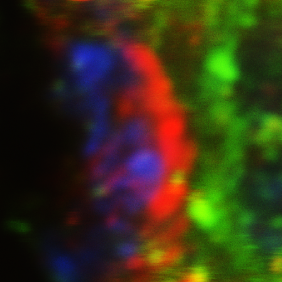

Supplement: Supplementary file 9 — EV Figure Source Data [file 44318_2024_74_MOESM9_ESM.zip › Figure EV2/EV2A/EV2A_Tet-ED-OE.tif]

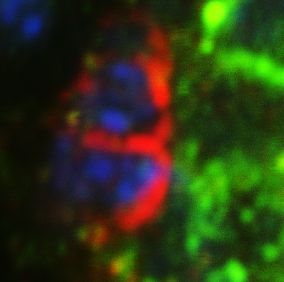

Supplement: Supplementary file 9 — EV Figure Source Data [file 44318_2024_74_MOESM9_ESM.zip › Figure EV2/EV2A/EV2A_Tet-KD1.tif]

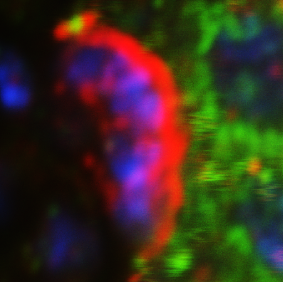

Supplement: Supplementary file 9 — EV Figure Source Data [file 44318_2024_74_MOESM9_ESM.zip › Figure EV2/EV2A/EV2A_Tet-WT-OE.tif]

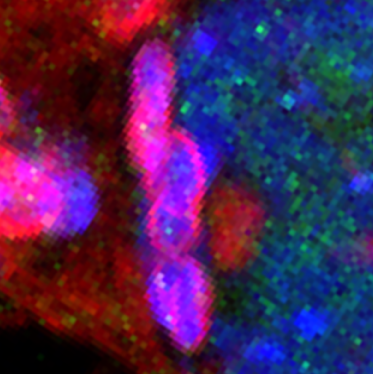

Supplement: Supplementary file 9 — EV Figure Source Data [file 44318_2024_74_MOESM9_ESM.zip › Figure EV3/EV3B/EV3B_luc-KD_Left.tif]

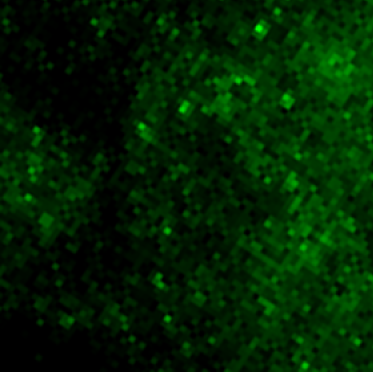

Supplement: Supplementary file 9 — EV Figure Source Data [file 44318_2024_74_MOESM9_ESM.zip › Figure EV3/EV3B/EV3B_luc-KD_Right.tif]

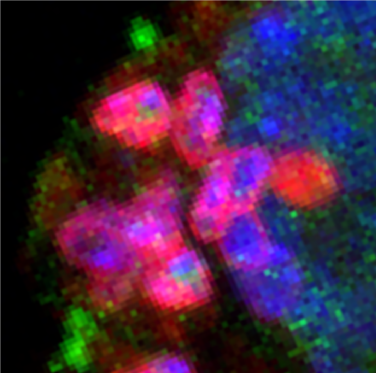

Supplement: Supplementary file 9 — EV Figure Source Data [file 44318_2024_74_MOESM9_ESM.zip › Figure EV3/EV3B/EV3B_Tet-KD1_Left.tif]

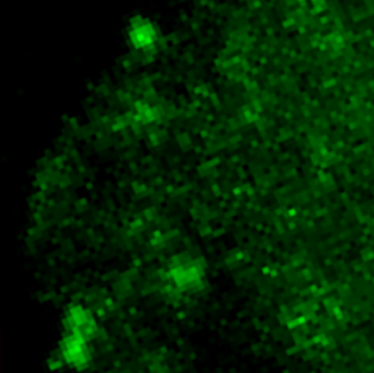

Supplement: Supplementary file 9 — EV Figure Source Data [file 44318_2024_74_MOESM9_ESM.zip › Figure EV3/EV3B/EV3B_Tet-KD1_Right.tif]

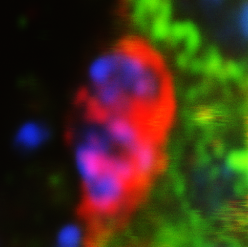

Supplement: Supplementary file 9 — EV Figure Source Data [file 44318_2024_74_MOESM9_ESM.zip › Figure EV4/EV4A/EV4A_Bap170-KD.tif]

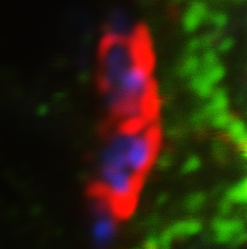

Supplement: Supplementary file 9 — EV Figure Source Data [file 44318_2024_74_MOESM9_ESM.zip › Figure EV4/EV4A/EV4A_Bap180-KD.tif]

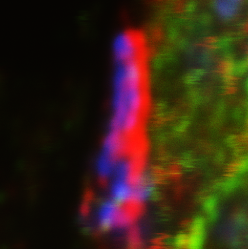

Supplement: Supplementary file 9 — EV Figure Source Data [file 44318_2024_74_MOESM9_ESM.zip › Figure EV4/EV4A/EV4A_brm-KD.tif]

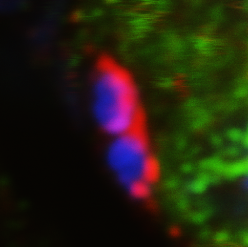

Supplement: Supplementary file 9 — EV Figure Source Data [file 44318_2024_74_MOESM9_ESM.zip › Figure EV4/EV4A/EV4A_luc-KD.tif]

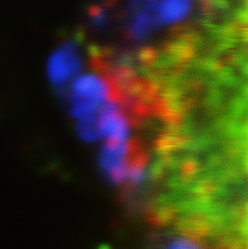

Supplement: Supplementary file 9 — EV Figure Source Data [file 44318_2024_74_MOESM9_ESM.zip › Figure EV4/EV4A/EV4A_osa-KD.tif]
