# Supplementary material for: Niche Tet maintains germline stem cells independently of dioxygenase activity
Source: EMBO J. 2024 Mar 18;43(8):9. doi: 10.1038/s44318-024-00074-9 (PMC11021519; doi:10.1038/s44318-024-00074-9)
Supplement: Supplementary file 11 — Expanded View Figures [file 44318_2024_74_MOESM11_ESM.pdf]

Expanded View Figures

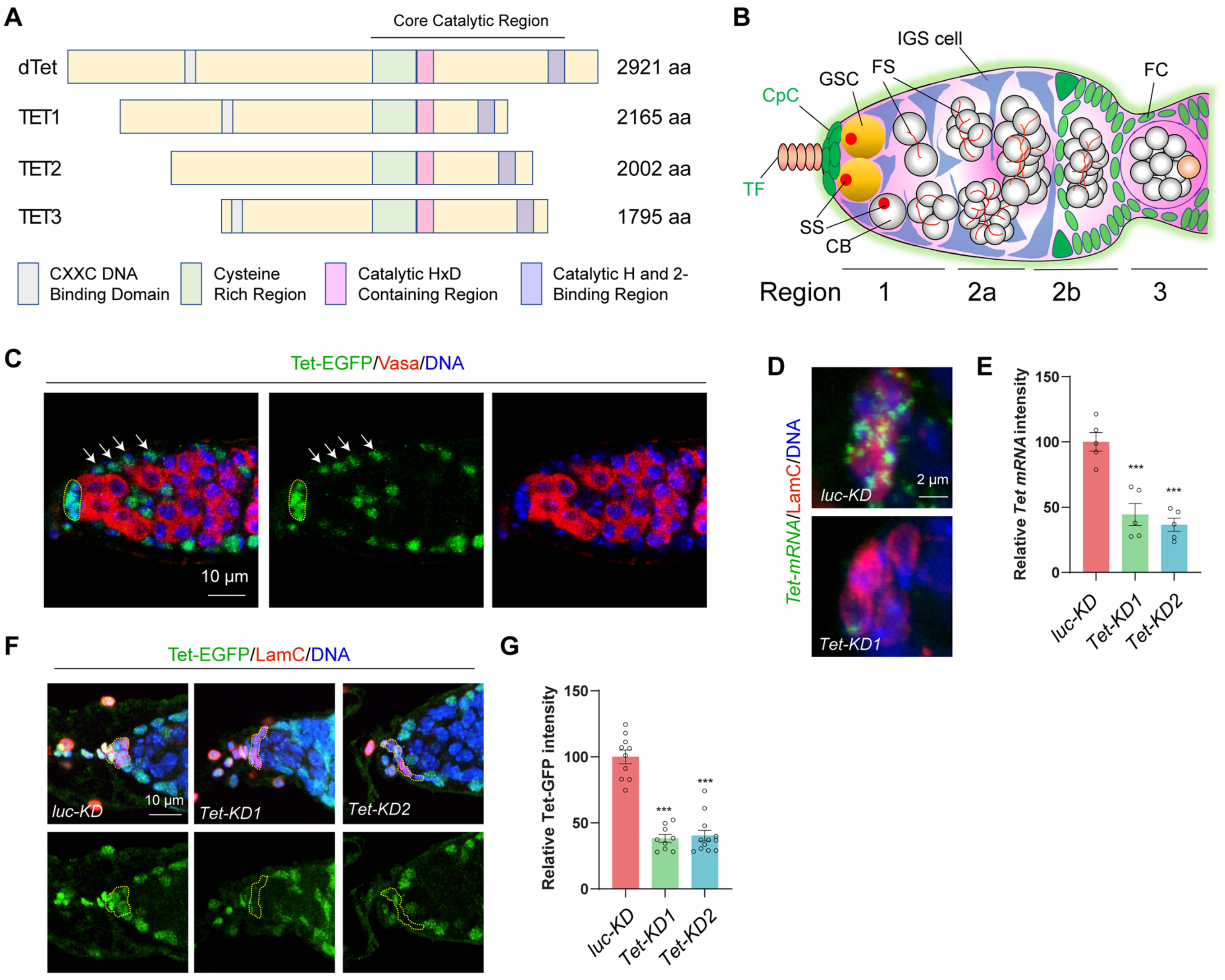

Figure EV1. Validating the knockdown efficiency of Tet RNAi lines.

(**A**) Domain architecture of *Drosophila* Tet (NP_001261344.1), human TET1 (NP_001393294.1), TET2 (NP_001120680.1), and TET3 (NP_001274420.1). They share the highly conserved CXXC DNA binding domain, the cysteine-rich region, and the catalytic domain regions. (**B**) A schematic diagram of a *Drosophila* germarium, which contains GSCs, cystoblasts (CBs), mitotic cysts (2-cell, 4-cell, and 8-cell cysts) and 16-cell cysts, and stage 1 egg chamber in region 1, 2a, 2b, and 3. Abbreviations: TF, terminal filament; CPC, cap cell; IGS cell, inner germarial sheath cell; CB, cystoblast, SS, spectrosome; FS, fusome; and FC, follicle cells. (**C**) Confocal images show that Tet-EGFP (arrows) does not express in Vasa-labeled (Red) germ cells. (**D–G**) *bab1*^{ts}-driven *Tet-KD* significantly reduces *Tet mRNA* (**D**) and Tet-EGFP (**F**) expression in niche cells. (**E**, **G**) Quantification results. Data information: In (**E** and **G**), data are presented as mean ± SEM. ***$P \leq 0.001$ (Student's t-test). *n* = number of germaria. Source data are available online for this figure.

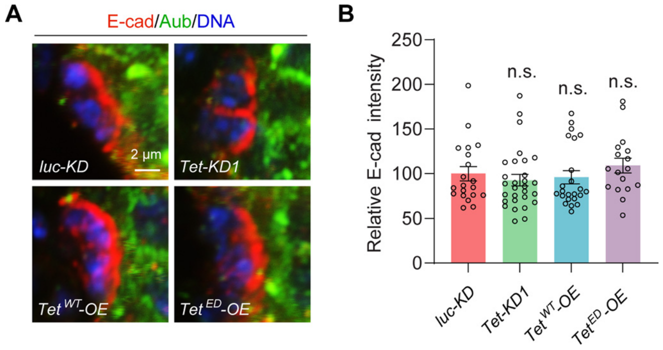

Figure EV2. Tet is dispensable in the niche for maintaining E-cadherin accumulation at the GSC-niche junction.

(**A**, **B**) *bab1*^{ts}-driven *Tet-KD* does not have an obvious effect on E-cadherin (E-cad) expression. The Argonaute/Piwi family protein, Aubergine (Aub), is enriched in germ cells. anti-Aub antibody was used to label germ cells. (**B**) Quantification results (n = number of germaria). Data are presented as mean ± SEM. n.s., no significance (Student's t-test). Source data are available online for this figure.

 © The Author(s)

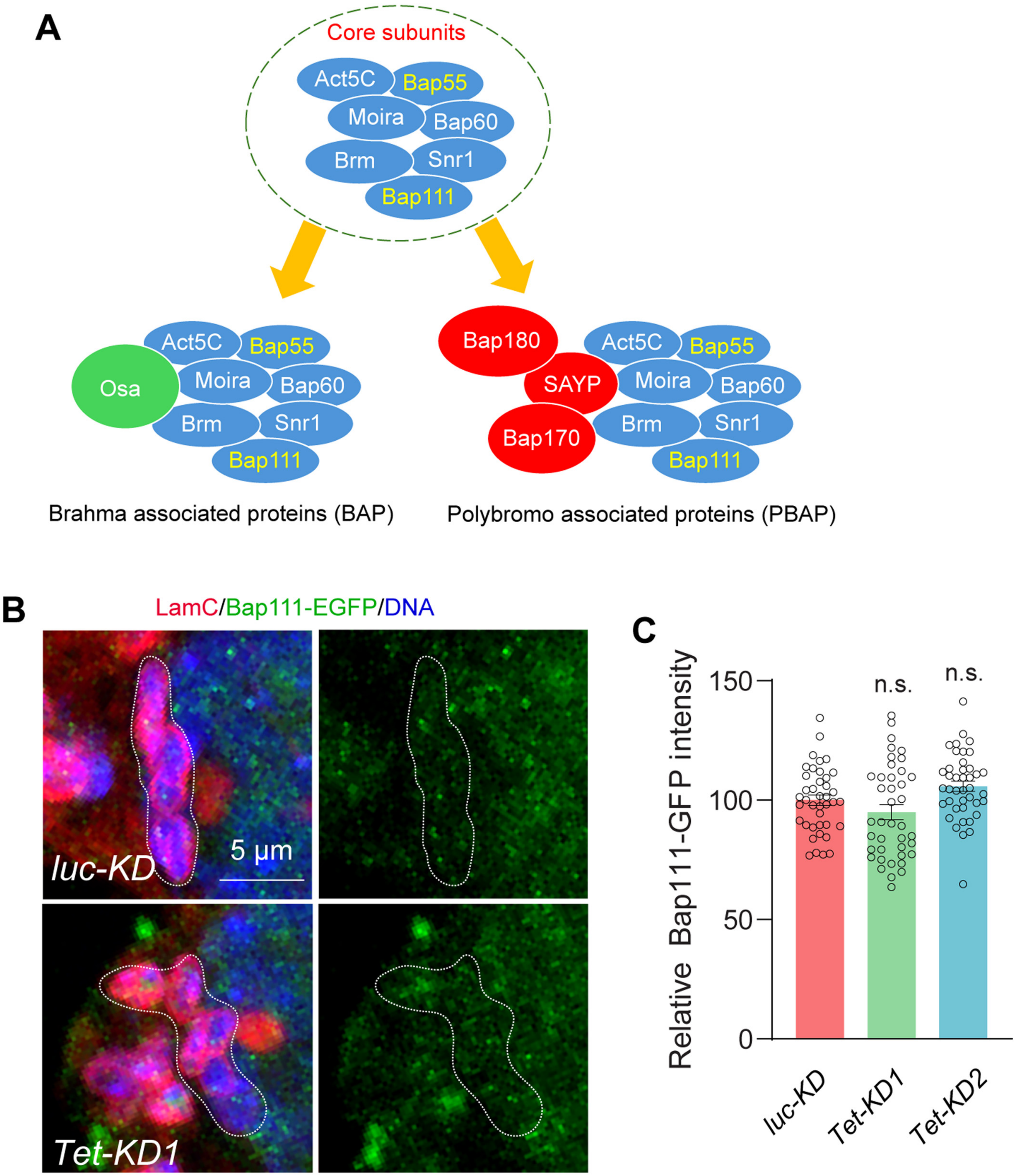

Figure EV3. Tet is dispensable in the niche for maintaining Bap111-EGFP expression.

(**A**) The schematic drawing of Brm complexes. Brm-containing chromatin remodelers can be divided into two types: BAP and PBAP according to their specific subunits, Osa and Bap170/180/SAYP, respectively (Hong and Choi, 2016). (**B, C**) *bab1*^{ts}-driven *Tet-KD* does not have an obvious effect on Bap111-EGFP expression in niche cells. (**C**) Quantification results (n = number of germaria). Data are presented as mean ± SEM. n.s., no significance (Student's t-test). Source data are available online for this figure.

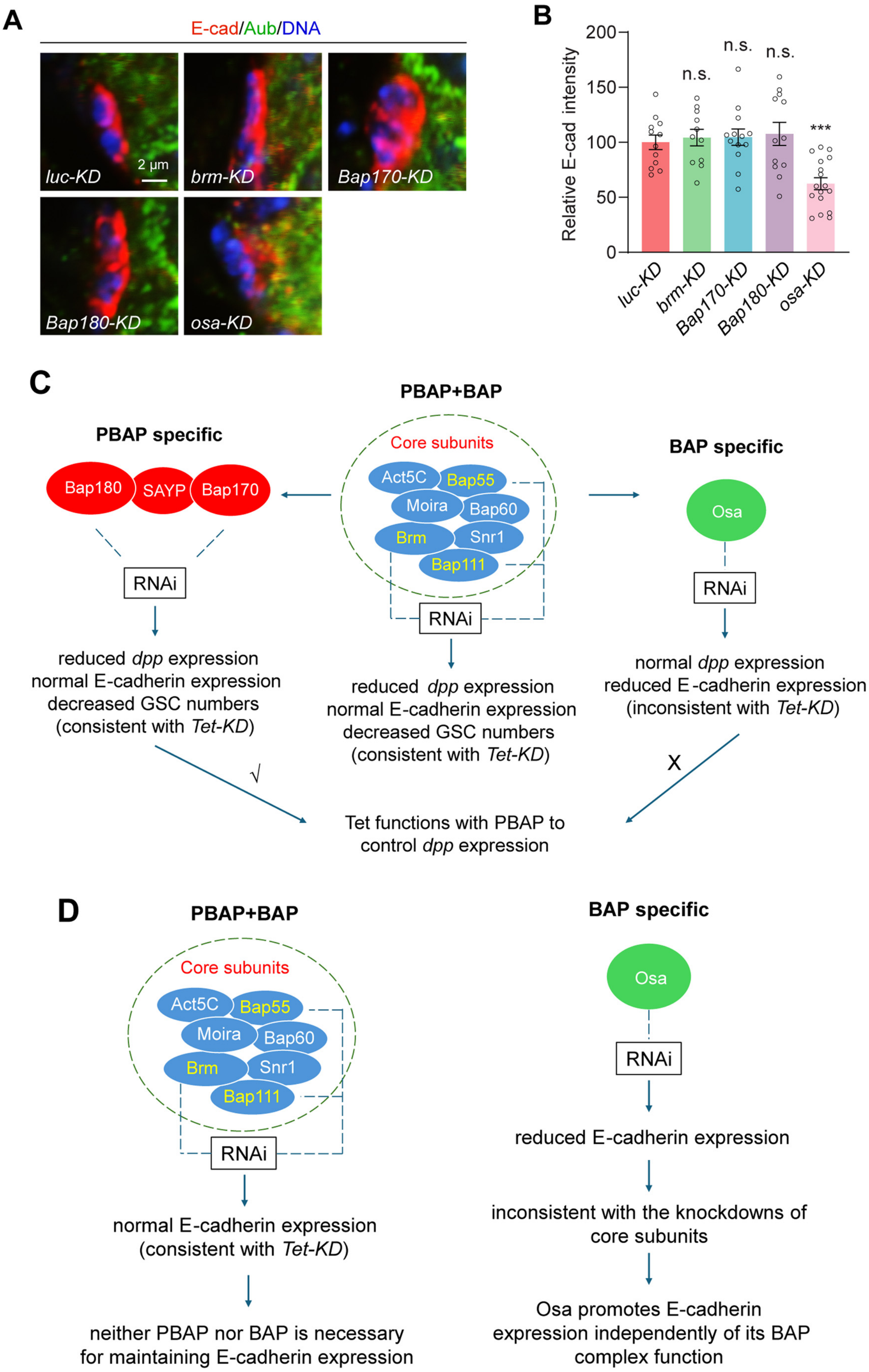
A
E-cad/Aub/DNA
luc-KD
2 µm
brm-KD
Bap170-KD
Bap180-KD
osa-KD
B
Relative E-cad intensity
200
150
100
50
0
n.s.
n.s.
n.s.
***
luc-KD
brm-KD
Bap170-KD
Bap180-KD
osa-KD
C
PBAP+BAP
PBAP specific
Core subunits
BAP specific
Bap180
SAYP
Bap170
Act5C
Bap55
Moira
Bap60
Brm
Snr1
Bap111
Osa
RNAi
RNAi
RNAi
reduced dpp expression
normal E-cadherin expression
decreased GSC numbers
(consistent with Tet-KD)
reduced dpp expression
normal E-cadherin expression
decreased GSC numbers
(consistent with Tet-KD)
normal dpp expression
reduced E-cadherin expression
(inconsistent with Tet-KD)
√
X
Tet functions with PBAP to
control dpp expression
D
PBAP+BAP
BAP specific
Core subunits
Act5C
Bap55
Moira
Bap60
Brm
Snr1
Bap111
Osa
RNAi
RNAi
normal E-cadherin expression
(consistent with Tet-KD)
neither PBAP nor BAP is necessary
for maintaining E-cadherin expression
reduced E-cadherin expression
inconsistent with the knockdowns of
core subunits
Osa promotes E-cadherin
expression independently of its BAP
complex function

 © The Author(s)

Figure EV4. Osa is required in the niche for E-cadherin expression.

(**A**, **B**) *bab1^ts^*-driven *brm-KD*, *Bap170-KD*, and *Bap180-KD* have no obvious effect on E-cadherin expression in cap cells, but *osa-KD* significantly decreases E-cad expression in niche cells. (**B**) Quantification results (n = number of germaria). Data are presented as mean ± SEM. n.s., no significance (Student's t-test). (**C**) Knockdowns of PBAP-specific but not BAP-specific subunits exhibit similar phenotypes compared to *Tet-KD* and core subunits-*KD*. (**D**) Knockdowns of core subunits have no effect on the expression of E-cadherin, indicating that neither PBAP nor BAP is required for maintaining E-cadherin expression. The reduced expression level of E-cadherin observed in *osa-KD* germaria is likely caused by the function of Osa independent of BAP complex. Further experiments need to be conducted in the future to verify this intriguing phenomenon. Source data are available online for this figure.

© The Author(s)
